# The effect of combined glutamate receptor blockade in the NTS on the hypoxic ventilatory response in awake rats differs from the effect of individual glutamate receptor blockade

**DOI:** 10.14814/phy2.12092

**Published:** 2014-08-08

**Authors:** Matthew E. Pamenter, Jetson Nguyen, John A. Carr, Frank L. Powell

**Affiliations:** 1 Division of Physiology, Department of Medicine, University of California San Diego, La Jolla, California; 2 Department of Zoology, University of British Columbia, Vancouver, British Columbia, Canada

**Keywords:** AMPA receptors, hypercapnic ventilatory response, kainate receptors, NMDA receptors

## Abstract

Ventilatory acclimatization to hypoxia (VAH) increases the hypoxic ventilatory response (HVR) and causes persistent hyperventilation when normoxia is restored, which is consistent with the occurrence of synaptic plasticity in acclimatized animals. Recently, we demonstrated that antagonism of individual glutamate receptor types (GluRs) within the nucleus tractus solitarii (NTS) modifies this plasticity and VAH (*J. Physiol*. 592(8):1839–1856); however, the effects of combined GluR antagonism remain unknown in awake rats. To evaluate this, we exposed rats to room air or chronic sustained hypobaric hypoxia (CSH, Pi_O2_ = 70 Torr) for 7–9 days. On the experimental day, we microinjected artificial cerebrospinal fluid (ACSF: sham) and then a “cocktail” of the GluR antagonists MK‐801 and DNQX into the NTS. The location of injection sites in the NTS was confirmed by glutamate injections on a day before the experiment and with histology following the experiment. Ventilation was measured in awake, unrestrained rats breathing normoxia or acute hypoxia (10% O_2_) in 15‐min intervals using barometric pressure plethysmography. In control (CON) rats, acute hypoxia increased ventilation; NTS microinjections of GluR antagonists, but not ACSF, significantly decreased ventilation and breathing frequency in acute hypoxia but not normoxia (*P *<**0.05). CSH increased ventilation in hypoxia and acute normoxia. In CSH‐conditioned rats, GluR antagonists in the NTS significantly decreased ventilation in normoxia and breathing frequency in hypoxia. A persistent HVR after combined GluR blockade in the NTS contrasts with the effect of individual GluR blockade and also with results in anesthetized rats. Our findings support the hypotheses that GluRs in the NTS contribute to, but cannot completely explain, VAH in awake rats.

## Abstract (precis)

Ventilatory acclimatization to hypoxia involves plasticity in the central nervous system, as well as in arterial chemoreceptors. NMDA and AMPA glutamate receptors in the NTS contribute to different aspects of ventilatory acclimatization to hypoxia. However, total ionotropic glutamate receptor blockade in the NTS does not block acclimatization and the effects are not predictable from those of individual antagonists.

## Introduction

Acute exposure to hypoxia stimulates arterial carotid body chemoreceptors, which in turn mediate a reflex increase in breathing (i.e., the hypoxic ventilatory response [HVR]) (Powell et al. 1998). When hypoxia is sustained for days to months (i.e., chronic sustained hypoxia [CSH]; e.g., during acclimatization to altitude), an additional time‐dependent increase in ventilation occurs, which has been termed ventilatory acclimatization to hypoxia (VAH) (Powell et al. 1998). Two mechanisms have been described to explain this increase in the HVR during CSH: (1) an increase in the oxygen sensitivity of carotid body glomus cells and (2) an increase in the central nervous system (CNS) responsiveness to carotid sinus nerve inputs (Bisgard and Neubauer 1995; Dwinell and Powell 1999; Wilkinson et al. 2010; Kumar and Prabhakar 2012). Considerable progress has been made toward understanding the mechanisms of enhanced carotid body chemosensitivity to oxygen during CSH, but the mechanism(s) that mediate increased CNS gain of the HVR are not well understood.

VAH persists after the removal of hypoxic stimulation, which is consistent with the occurrence of synaptic plasticity within the ventilatory control circuits (Bisgard and Neubauer 1995; Powell et al. 1998). This suggests that the mechanism of CNS gain of the HVR in VAH involves synaptic modification. In the CNS, carotid body afferent neurons project to the nucleus of the solitary tract (nucleus tractus solitarii [NTS]) (Lipski et al. 1977; Housley and Sinclair 1988). There is considerable evidence supporting glutamate as the main excitatory neurotransmitter from the carotid sinus nerve to the NTS, and as a key mediator of the acute HVR. Specifically, (1) in response to a hypoxic stimulus, glutamate is released into the NTS coincident with the HVR (Mizusawa et al. 1994; Richter et al. 1999), (2) glutamate injection into the NTS mimics the HVR (Mizusawa et al. 1994), (3) both the HVR and hypoxia‐mediated glutamate release are prevented by carotid body denervation (Mizusawa et al. 1994), (4) NTS microinjection or systemic injection of glutamatergic *N*‐methyl‐d‐aspartate receptor (NMDAR) antagonists attenuates the HVR in a variety of species (Vardhan et al. 1993; Mizusawa et al. 1994; Soto‐Arape et al. 1995; Ohtake et al. 1998; Harris and Milsom 2001; Tarakanov et al. 2004; Reid and Powell 2005; Pamenter et al. 2014), while (5) NTS microinjection of a broad‐spectrum excitatory amino acid antagonist further attenuates responses to short‐term hypoxia beyond the effect of NMDAR blockade alone (Mizusawa et al. 1994); (6) simultaneous antagonism of NMDARs and glutamatergic *α*‐amino‐3‐hydroxy‐5‐methyl‐4‐isoxazolepropionic acid receptors (AMPARs) within the NTS reduce ventilatory and cardiac responses to chemical or CO_2_‐mediated carotid body stimulation in anesthetized and awake rats (Vardhan et al. 1993; Zhang and Mifflin 1993; Haibara et al. 1999; Braga et al. 2007).

The role of glutamate neurotransmission in VAH is less clear. The effect of systemic NMDAR blockade on the HVR is changed by CSH (Reid and Powell 2005), and NMDAR mRNA and protein expression increase following 2 weeks of CSH in murine medulla (El Hasnaoui‐Saadani et al. 2007), while NMDAR NR1 and AMPAR GluR1 subunit phosphorylation increase 35% and 70%, respectively, in CSH‐conditioned rat NTS relative to CON rats (Pamenter et al. 2014). In a recent study, we found different effects of individually blocking either AMPARs or NMDARs in the NTS on VAH in awake unrestrained rats (Pamenter et al. 2014). NMDAR blockade in the NTS of chronically hypoxic rats completely blocked the acute HVR but did not reverse the increased ventilation in normoxia associated with acclimatization. In contrast, AMPAR blockade in the NTS reduced ventilation under all conditions in which it was stimulated, that is, during acute hypoxia in control and in acute normoxia in chronically hypoxic rats. This effect was specific to hypoxia, however, as the ventilatory response to hypercapnia was not affected by AMPAR blockade in the NTS. These results suggest that there may be complex interactions between different types of glutamate receptors in chronically hypoxic animals when glutamate is released in the NTS by carotid body stimulation. In the hippocampus, glutamate receptors interact via a variety of direct ionotropic effects and indirect nonionotropic cross‐talk mechanisms such that modulation of one receptor subtype can either up‐ or downregulate the function of the other subtype (Bai et al. 2002). Therefore, we hypothesized that the effect of comprehensive blockade of ionotropic glutamate receptors in the NTS on VAH could differ from blocking only one type of glutamate receptor at a time. To test this, we examined the effect of NTS‐specific microinjections of a cocktail of antagonists targeted to the three known types of ionotropic glutamatergic receptors (i.e., AMPARs, NMDARs, and kainate receptors [KARs]) on the acute HVR and VAH in conscious rats acclimatized to sea level or hypobaric hypoxia (Pi_O2_ = 70 mmHg) for 7–9 days.

## Materials and Methods

### Experimental animals and surgery

All surgical procedures and protocols were performed in accordance with the relevant guidelines of The University of California San Diego *Institutional Animal Care and Use Committee*. Male Sprague–Dawley rats (Charles River) weighing 250–300 g were housed under a 12:12 h light:dark cycle and fed a standard diet ad libitum.

Animals were randomly divided into two experimental groups: (1) normoxic sea level controls (CON, *n* = 7) and (2) chronically hypoxic rats (CSH, *n* = 9). The CSH‐conditioned rats were acclimatized to a simulated altitude of 5500 m in a hypobaric chamber at 380 Torr (Pi_O2_ = 70 Torr, equivalent to ~10% O_2_ at sea level barometric pressure) for 7–9 days. The chamber was returned to sea level for 15 min every 3–4 days for general cage maintenance or when it was necessary to remove animals for experimentation. At least 2 days prior to acclimatization in normoxia or CSH, all animals underwent surgery for implantation of guide cannulae, arterial catheters, and body temperature telemetry probes.

All surgeries were performed under isoflurane anesthesia (initially 5% isoflurane in 100% O_2_ and maintained at 2–3% isoflurane). Stereotaxic surgery (Kopf Instruments, Tujunga, CA) was used to implant a stainless steel guide cannula (Plastics One, Roanoke, VA) bilaterally into the NTS to deliver pharmacological agents. Two holes were drilled into the cranium into which screws would fit firmly and the guide cannula was secured to the skull using acrylic resin that fixed the guide cannula to these two screws. The microinjection needle was 1 mm longer than the guide cannula and projected into the NTS (target coordinates: AP = 0.3 mm [from obex], ML = 0.7 mm, DV = 0.5 mm; Housley and Sinclair 1988).

Arterial catheters were inserted through the femoral artery and reached the abdominal aorta of the rat for blood gas sampling. Polyethylene tubing (PE‐50) was heated and stretched to fit the diameter of the artery, and sutured in place. The stretch allowed for a single tube with no joints that was more resistant to the formation of blood clots. The catheter was tunneled beneath the dorsal skin and exited at the back of the neck through a stainless steel headbutton that was sutured in place for easy access. A stainless steel ring was screwed to the headbutton to protect the catheter.

A telemetry thermometer probe (Emitter, Respironics, Bend, OR) was implanted to monitor body temperature. The body temperature is required for an accurate calculation of the tidal volume (Vt, see below). The emitter was implanted into the abdominal cavity and sutured in place to the interior wall of the abdomen.

### Plethysmography

Inspired ventilation (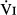) was measured using barometric pressure method of plethysmography modified for continuous flow (Jacky 1978). On the day of experimentation, individual animals were sealed into a 7 L Plexiglas chamber. An electronic gas mixer (MFC‐4, Sable Systems, Las Vegas, NV) was used to supply the animal with an inflowing gas mixture (3 L/min) of controlled O_2_ and CO_2_ (balance N_2_). Inflowing gas entered the chamber through a tube (7 cm long and 1 cm in diameter) that was filled with smaller PE‐50 tubing of similar length to create a high‐impedance input and reduce the loss of pressure signals. Gas exited the plethysmograph chamber through a high‐impedance valve to a vacuum pump (Dayton Electric, Chicago, IL). Pressure inside the box was referenced to atmospheric pressure using a water manometer. Atmospheric pressure corrected for standard gravity and room temperature was measured on each experimental day. To ensure a controlled gas mixture in the chamber, the pressure inside the chamber was positive (<0.5 cm H_2_O) with a small leak from the chamber (20 ga needle) that also prevented slow changes in baseline with temperature changes. Chamber gas concentrations were measured using a mass spectrometer (Perkin‐Elmer 1100 Medical Gas Analyzer, Pomona, CA) that was calibrated for O_2_ and CO_2_ on each experimental day. A chamber temperature probe (Thermalert TH‐5, Physitemp, Clifton, NJ) was sealed inside the box, and a humidity probe was placed into the box through a hole that was cut and sealed for this purpose. Inspiration produces humidity‐ and temperature‐related changes in pressure that can be monitored with a differential pressure transducer (DP45, Validyne, Northridge, CA) referenced to atmosphere. Output from the transducer was recorded on a digital data acquisition system (see below). Respiratory frequency (fr) was calculated directly from the ventilation‐induced pressure swings. Tidal volume (Vt) was calculated from the ventilation‐induced pressure changes using an equation from Drorbaugh and Fenn (1955) and modified for flow‐through plethysmography by Jacky (1978). Prior to each experiment, calibration pulses (0.2, 0.5, and 1.0 mL) were generated using a gas‐tight syringe by injecting air pulses into the chamber at a rate similar to the rats' inspiratory time. The amplitude of calibration pulses varied <10% for injections lasting between 100 and 350 msec (corresponding to inspiratory times).

Ventilation was determined under poikilocapnic conditions. The animals were given at least 40 min to habituate to the plethysmograph at their chronic inspired O_2_ levels before study. Two inspired O_2_ levels (Fi_O2_ = 10 and 21%) were used in the study. 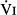 was measured between 10 and 15 min after the rats were exposed to a new inspired gas concentration by selecting a stable period of data without movement artifacts or sighs.

### Arterial blood gas measurements

Single arterial batch samples (0.2 mL) were taken while the rats were breathing 10% or 21% O_2_ (*n *=**3 for each group), as well as 7% CO_2_ in 21% O_2_ (*n *=**3–5 for each group). Samples were obtained after control and drug microinjections. These samples were corrected for body temperature and analyzed (Instrumentation Laboratory Gem Premier 5000, Lexington, MA) for Pao_2_, Paco_2_, and arterial pH.

### Microinjections

**Figure 1.**
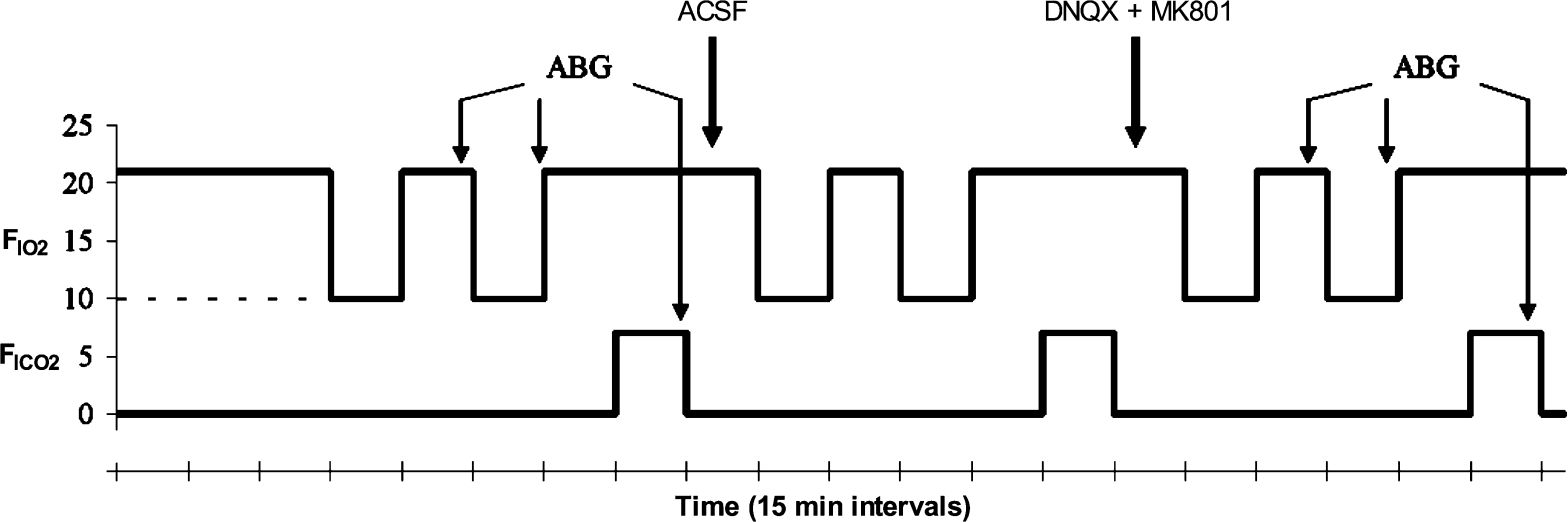
Experimental protocol. Schematic diagram indicates changes in inspired gas concentrations during experiments and experimental protocol. Animals were placed in the plethysmograph for 25–45 min at their chronic inspired O_2_ level prior to the start of an experiment to allow time to acclimate to the environment. Solid line indicates control (CON) rats (21% O_2_); dashed line indicates chronic sustained hypoxia (CSH) rats (10% O_2_). Arterial blood gas (ABG) samples were collected at the indicated time points. Animals received injections of artificial cerebral spinal fluid (ACSF: sham) and cocktail (DBQX + MK801) at the times indicated.

A microinjection needle made to fit the guide cannula was connected to a 500 nL Hamilton microsyringe through a polyethylene tube. The microinjection needle projected 1 mm beyond the guide cannula into the NTS. The polyethylene tube ran through the lid of the plethysmograph and was sealed. After control ventilatory measurements were made at 10 and 21% O_2_, 50 nL of artificial cerebrospinal fluid (ACSF, in mmol/L: 115 NaCl, 2.0 KCl, 2.2 KH_2_PO_4_, 25 NaHCO_3_, 10 d‐glucose, 1.2 MgSO_4_, and 2.5 CaCl_2_, adjusted to pH 7.4 with HCl) (Youssef et al. 2001), or a “cocktail” of the NMDAR antagonist (+)‐5‐methyl‐10,11‐dihydro‐5H‐dibenzo[a,d]cyclohepten‐5,10‐imine hydrogen maleate (MK‐801, 2.5 nmol/L) and the AMPAR/KAR antagonist 6,7‐dintitroquinoxaline‐2,3‐dione (DNQX, 1.0 *μ*mol/L) dissolved in ACSF was injected into the NTS. The pharmacological doses used in the present study are sufficient to inhibit physiological responses to NTS‐mediated stimulation throughout the time frame of our experiments in awake or anesthetized rats, in accordance with studies in similar preparations from other laboratories (Florentino et al. 1990; Ohta et al. 1993; Mizusawa et al. 1994; Machado et al. 2000). Following the injection, ventilatory measurements were collected again in each gas mixture. All animals received both ACSF and drug injections and ventilation was examined after each injection. The experimental protocol was as per the schematic diagram (Fig. 1) and animals were permitted at least 1 h to rest in their home cages between the completion of sampling following the first injection and receiving the second injection. Animals received ACSF injections before drug injections and experimental protocols were not randomized because the effects of these drugs take hours to days to wash off. In some experiments, animals received a second injection of ACSF in place of cocktail microinjection to assess the impact of the experimental time course. No differences were observed between the effects of ACSF in multiple trials in the same animal (data not shown). Ventilatory effects of the drugs were determined by comparing the data obtained following the control measurements with those collected following the ACSF or ACSF + drug microinjection. All drugs were purchased from Sigma‐Aldrich (St. Louis, MO).

### Histology and localization of microinjections

**Figure 2.**
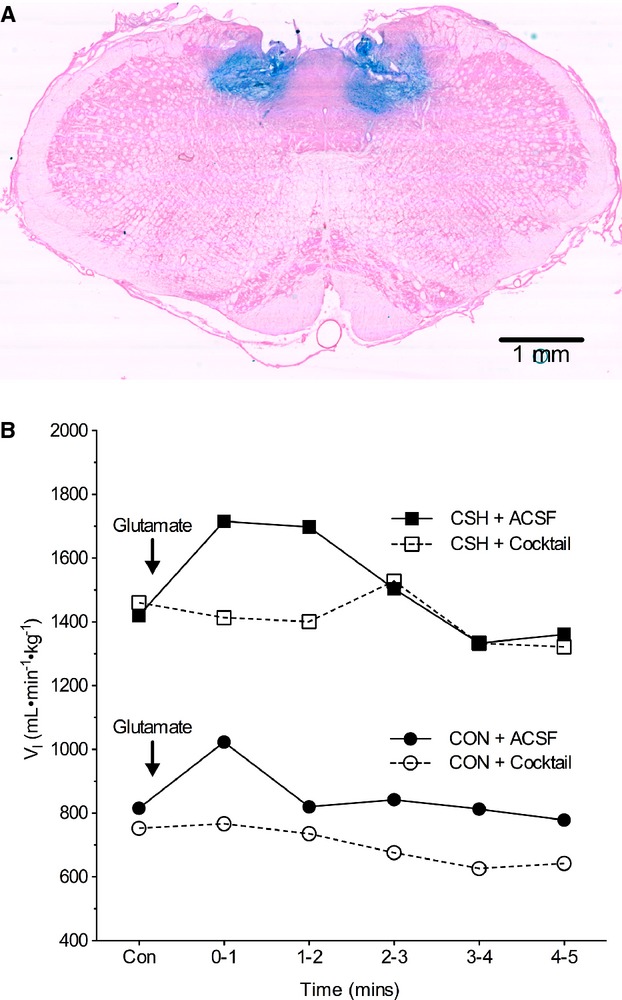
Location of NTS microinjection site. (A) Coronal section of the brainstem of a rat showing the sites of bilateral microinjections of Evan's blue, which is within the 500 *μ*m target area of the caudal NTS where carotid body afferents have a primary synapse and a ventilatory response to glutamate microinjection is evoked. Magnification 6.3×. (B) Sample time course of ventilatory responses to 50 nL glutamate microinjections in two different rats before and after cocktail microinjection. Animals were not used for further measurements or analysis if they showed no response to glutamate or if the cocktail did not block the effects of glutamate.

Colloidal gold or Evan's blue microinjections were used to localize the microinjection sites (Fig. 2A). At the end of the experiment, colloidal gold (50 nL) or Evan's blue (50 nL) was microinjected through the guide cannulae into the NTS at the same site as the drug delivery. The animals were anesthetized with an overdose of sodium pentobarbital and transcardially perfused with ice‐cold ACSF followed by 4% paraformaldehyde. The brainstem was removed and postfixed in 2% paraformaldehyde for 1 day, and then stored in sucrose (30%). The brainstems were frozen in isopentane (−140°C) and sectioned (slice thickness = 30–50 *μ*m) on a cryostat (Cryocut 1800, Leica Biosystems, Wetzlar, Germany). For animals microinjected with the colloidal gold, stains were enhanced using silver intensification solution (Ted Pella Inc., Redding, CA), which marked the microinjection sites with black staining. Aqueous eosin Y was used as a cytoplasmic counterstain. On a day before they were studied with the full protocol, rats received test glutamate microinjections before and after cocktail microinjections (Fig. 2B). For data analysis, we only used animals that had a positive response to glutamate that was prevented by cocktail microinjection, and in which the microinjection site was located within 500 *μ*m of the NTS target. We have shown previously that no ventilatory responses are observed if the histological data shows a microinjection that is more than 500 *μ*m away from the commissural caudal NTS target site, but similar responses are obtained if microinjections are localized within 500 *μ*m of the target (Pamenter et al. 2014).

### Data collection and analysis

Ventilatory data from the plethysmograph was digitized with an analog‐digital converter (National Instruments E‐Series 16 channel with 16‐bit multiplexing) and custom software (LabVIEW SignalExpress; National Instruments, Austin, TX). Digitized data were analyzed with custom software (UCSD Division of Physiology Plethysmography Toolbox running on The Mathworks MATLAB, Natick, MA). The pressure signal was analyzed for amplitude (with corrections for changes in the baseline during a breath to calculate inspired tidal volume [Vt] as described earlier), timing to calculate respiratory frequency (fr), and the duration of inspiration and expiration (Ti and Te, respectively). At least 10 sets of breaths comprising at least 10 consecutive breaths were selected for analysis from the final 5 min of each experimental treatment epoch and were analyzed for each period. Breaths were chosen from periods where the animal was awake but not active (i.e., not only actively grooming or exploring but also not asleep, as determined by visual examination).

Statistical analysis was performed using commercial software (SPSS 15.0, SPSS Inc., Chicago, IL). For all experiments, individual *n* values correspond to a single animal treated with CON or CSH acclimatization protocols and then treated with sham or cocktail, as described earlier, before and after exposure to 21% O_2_ and then either acute hypoxia (10% O_2_) or acute hypercapnia (7% CO_2_). Values are presented as mean ± SEM. *P *<**0.05 was considered statistical significance. All data were normally distributed (Shapiro–Wilk: *P *>**0.05) with equal variance (*P *>**0.05). For the drug microinjection data, a three‐way repeated measures analysis of variance (ANOVA) was used to determine whether there was a statistically significant difference between the three independent factors considered: (1) the acute hypoxia (Fi_O2_ of 0.10 vs. 0.21); (2) the chronic oxygen condition (CON vs. CSH); and (3) the treatment (control [ACSF] vs. drug microinjection). If there was a significant three‐way interaction, the two‐way ANOVA was applied to the CON and CSH groups independently to test for significant interactions between Fi_O2_ and drug, and Bonferonni post hoc tests to determine significance between the independent variables or the change in the HVRs (Δ's). Bonferonni post hoc multiple comparisons tests were run on each of the dependent variables to compare the single point means of interest. The dependent variables analyzed were total inspired minute ventilation (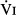), respiratory frequency (fr), tidal volume (Vt), inspiratory time (Ti), expiratory time (Te), average inspiratory flow rate (i.e., inspiratory drive; Vt/Ti) as an index of the ventilatory drive to breath, ΔHVR, and arterial Po_2_, Pco_2_, and pH.

## Results

### Acute and chronic hypoxia, but not ACSF microinjections increased ventilation

**Figure 3.**
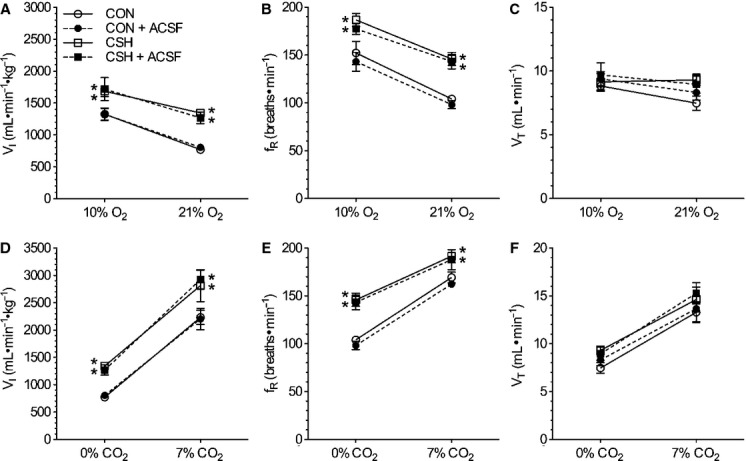
Effects of chronic hypoxia and sham acute ACSF injections on ventilatory responses to O_2_ and CO_2_. Effect of hypoxia and ACSF microinjections on (A) total minute ventilation (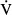
i), (B) breathing frequency (fr), and (C) tidal volume (Vt). Effect of hypercapnia and ACSF microinjections on 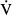
i (D), fr (E), and Vt (F). Data are mean ± SEM from *n *=**7 CON and 9 CSH rats. Asterisks (*) indicate significant (*P* < 0.05) difference between CON and CSH values.

The effects of acute hypoxia on ventilation were not changed by ACSF before or after CSH (Fig. 3), and the effects of CSH were similar to those reported previously (Aaron and Powell 1993; Reid and Powell 2005). Acute hypoxia (10% O_2_) increased 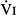 by 555 and 342 mL min^−1^ kg^−1^ in CON and CSH‐conditioned rats, respectively (Fig. 3A). These increases were predominately due to increased fr (Fig. 3B), while Vt was not significantly altered by hypoxia (Fig. 3C). The plots of 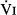 versus Fi_O2_ shifted upward with CSH and were roughly parallel to CON data such that 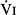 was increased by 570 and 360 mL min^−1^ kg^−1^ in normoxia and acute hypoxia, respectively, in CSH‐conditioned rats relative to CON rats. The increase in 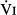 was due to increases in fr and Vt of CSH relative to CON (Fig. 3A–C). Ventilatory measurements in animals receiving ACSF‐microinjections varied from untreated control experiments in the same animals by <10% and these changes were not statistically significant.

Animals were also exposed to hypercapnia (7% CO_2_) as a control experiment to determine whether effects of ACSF or drugs were specific to hypoxic responses or affected ventilatory responses to other chemoreceptor stimuli. In sham‐treated animals, hypercapnia increased 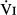 by threefold in CON animals and 2‐ to 2.5‐fold in CSH‐conditioned animals, and these increases were markedly greater than those induced by hypoxia. Elevated 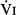 during hypercapnia was the product of significant increases in both fr and Vt in all treatment groups (Fig. 3D–F). As was the case for the HVR, the HCVR shifted parallel upward in CSH‐conditioned rats relative to CON rats.

### Combined glutamate receptor antagonism depressed ventilation in CON and CSH‐conditioned rats breathing acute hypoxia and in CSH‐conditioned rats breathing acute normoxia

**Figure 4.**
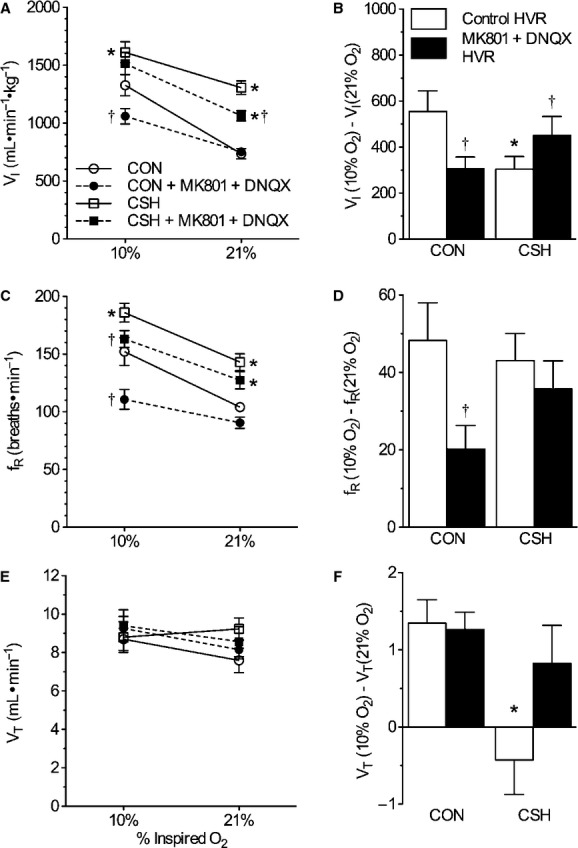
Effect of ionotropic glutamate receptor blockade in the NTS on the hypoxic ventilatory response (HVR) in normoxic control (CON) versus chronically hypoxic (CSH) rats. Effects of chronic hypoxia and MK801 + DNQX on (A) total minute ventilation (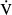
i) of CON and CSH rats before and after microinjection of drugs; (B) the magnitude of the HVR of 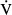
i (C) breathing frequency (fr) of CON and CSH rats before and after microinjection of drugs; (D) the magnitude of the HVR of fr; (E) tidal volume (Vt) of CON and CSH rats before and after microinjection of drugs; (F) the magnitude of the HVR of Vt. Data are mean ± SEM from *n *=**7 CON and 9 CSH rats. Asterisks (*) indicate significant (*P* < 0.05) difference between CON and CSH values. Daggers (†) indicate significant effect of drug relative to ACSF microinjection.

**Figure 5.**
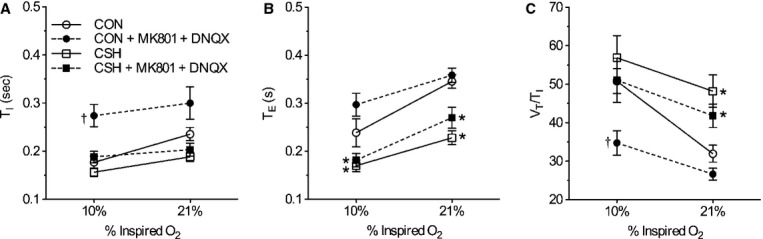
Effect of ionotropic glutamate receptor blockade in the NTS on the pattern of breathing and inspiratory drive in response to acute hypoxia in normoxic control (CON) versus chronically hypoxic (CSH) rats. Effects of chronic hypoxia and MK801 + DNQX on (A) inspiratory time (Ti), (B) expiratory time (Te), and (C) inspiratory drive (Vt/Ti) of CON and CSH rats before and after microinjection of drugs. Data are mean ± SEM from *n *=**7 CON and 9 CSH rats. Asterisks (*) indicate significant (*P* < 0.05) difference between CON and CSH values. Daggers (†) indicate significant effect of drug relative to ACSF microinjection.

Analysis with a three‐way ANOVA indicated a significant interaction between CON and CSH groups, 10% and 21% inspired Po_2_, and drug versus sham treatment. This evaluation was followed up with a two‐way ANOVA, which indicated that the interaction between cocktail microinjections in the NTS and acute inspired oxygen concentration on ventilation was significant in both CON and CSH‐conditioned animals (*P *=**0.04 and 0.03, respectively; Fig. 4A); post hoc analysis indicated that in CON rats the interaction between cocktail and acute inspired oxygen concentration was because of a significant difference with cocktail injection at 10% O_2_, but not at 21% O_2_; conversely, in CSH‐conditioned rats the interaction was because of a significant difference with cocktail injection at 21% O_2_, but not at 10% O_2_ (*P *<**0.05). Specifically, in CON rats, the cocktail had no effect on 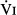 during normoxia but reduced 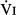 by 307 mL min^−1^ kg^−1^ in acute hypoxia (Fig. 4A). Consequently, the slope of the poikilocapnic HVR was depressed by the cocktail in CON rats (Fig. 4A and B). The decrease in 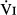 of CON rats was due to a 41.5 bpm drop in fr during acute hypoxia (*P *=**0.015; Fig. 4C and D), whereas Vt tended to increase slightly (ns; Fig. 4E and F). The cocktail‐mediated decrease in fr was due to increases in both Ti and Te in acute hypoxia, although only the change in Ti reached statistical significance (*P *=**0.015; Fig. 5A and B). The cocktail markedly decreased ventilatory drive (Vt/Ti) by 32% in CON animals breathing acute hypoxia (*P *<**0.001) and the slope of the ventilatory drive was decreased following drug administration (*P *<**0.006), reflecting a larger reduction in the inspiratory drive (Vt/Ti) in acute hypoxia in CON rats treated with the cocktail relative to CON rats treated with ACSF (Fig. 5C).

In CSH‐conditioned animals, the cocktail reduced 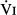 by 243 mL min^−1^ kg^−1^ in acute normoxia but the decrease was not significant in hypoxia (Fig. 4A). Consequently, the slope of the HVR was increased in CSH‐conditioned rats by the cocktail (Fig. 4A and B). The cocktail also decreased fr by 22.9 bpm in CSH‐conditioned animals breathing acute hypoxia (*P *=**0.009), but this effect was not apparent in total ventilation (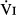) due to opposing effects of acute hypoxia on Vt in CSH‐conditioned animals treated with ACSF versus the cocktail. GluR antagonism did not have significant effects on other ventilatory parameters examined.

### Combined glutamate receptor antagonism did not reduce the HCVR or affect arterial blood gases

**Figure 6.**
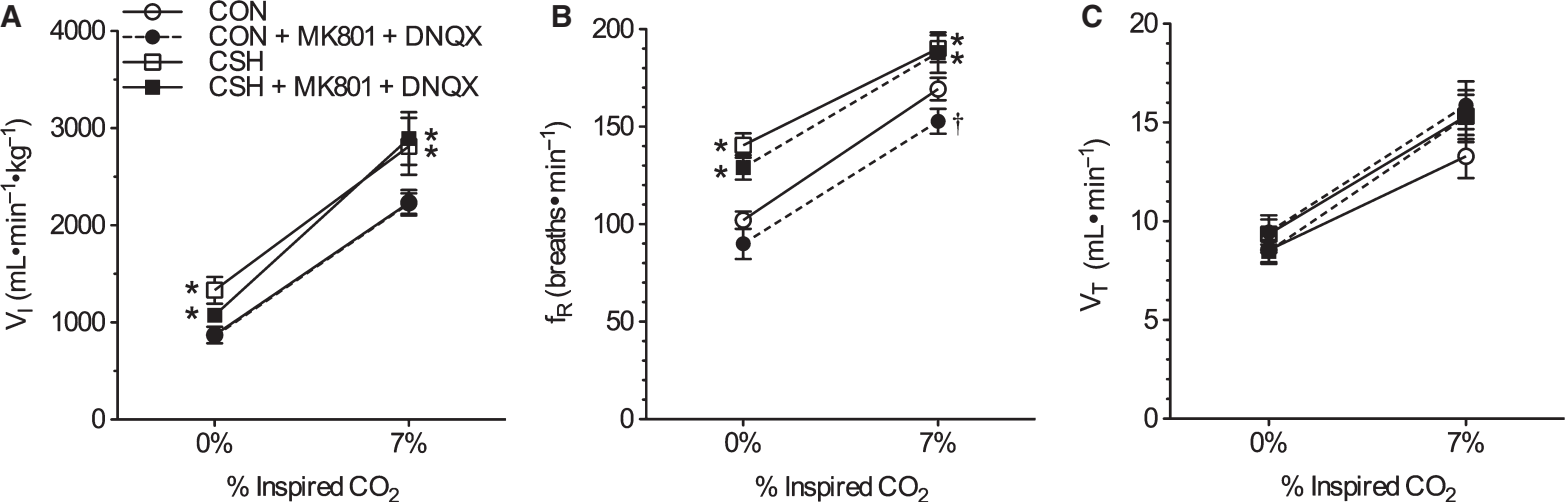
Effect of ionotropic glutamate receptor blockade in the NTS on the hypercapnic ventilatory response in normoxic control (CON) versus chronically hypoxic (CSH) rats. Effects of acute hypercapnia (7% CO_2_) and MK801 + DNQX on (A) total minute ventilation (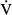
i), (B) breathing frequency (fr), and (C) tidal volume (Vt) of CON and CSH rats before and after microinjection of drugs. Data are mean ± SEM from *n *=**7 CON and 9 CSH rats. Asterisks (*) indicate significant (*P* < 0.05) difference between CON and CSH values. Daggers (†) indicate significant effect of drug relative to ACSF microinjection.

**Figure 7.**
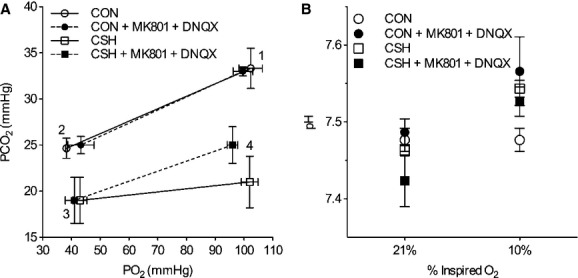
Arterial blood gases are not changed by ionotropic glutamate receptor blockade in the NTS. (A) Average changes in Po_2_ and Pco_2_ in response to microinjection of MK801 + DNQX into the NTS of normoxic control (CON) and chronically hypoxic (CSH) rats breathing acute levels of 10% and 21% O_2_. (B) Average changes in arterial pH in response to microinjection of MK801 + DNQX into the NTS of CON and CSH rats breathing acute levels of 10% and 21% O_2_. All blood gases are corrected for body temperature. Numbers refer to the following: (1) normoxic rats breathing 21% O_2_; (2) normoxic rats breathing 10% O_2_; (3) chronically hypoxic rats breathing 10% O_2_; (4) chronically hypoxic rats breathing 21% O_2_. Data are mean ± SEM from *n *=**3 rats per group.

Overall, there was not a significant interaction for 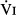 between acute inspired CO_2_% and drug treatment (Fig. 6). Arterial blood gases were examined in a subset of animals from each treatment group (Fig. 6, *n* = 3 for each group). Paco_2_ was depressed and Pao_2_ was increased in CSH‐conditioned rats relative to CON animals, as expected for ventilatory acclimatization to hypoxia (Olson and Dempsey 1978). Arterial pH was similar in both groups despite the lower Paco_2_ with CSH, as expected for metabolic compensation of the respiratory alkalosis (Olson and Dempsey 1978). Microinjection of the cocktail in the NTS had no significant effects on arterial blood gases or pH values (Fig. 7A and B).

## Discussion

We confirm a role for glutamatergic receptors in the NTS of rats in the hypoxic ventilatory response and ventilatory acclimatization to hypoxia. As discussed in the Introduction section and other publications, glutamate is released into the NTS from the carotid sinus nerve (Lipski et al. 1977; Housley and Sinclair 1988; Mizusawa et al. 1994; Richter et al. 1999) and activates local ionotropic glutamate receptors (GluRs), including AMPARs, NMDARs, and KARs, as well as metabotropic glutamate receptors (mGluRs) (Housley and Sinclair 1988; Connelly et al. 1992; Ohtake et al. 1998; Richter et al. 1999; Harris and Milsom 2001; Tarakanov et al. 2004; Reid and Powell 2005; Pamenter et al. 2014). Recently, we showed different effects of NMDAR versus AMPAR blockade in the NTS on the HVR and on VAH (Pamenter et al. 2014). Here, we show that the effect of combined ionotropic GluR blockade is different from the effects of blocking individual types of GluRs. Also, our results in awake rats differ from the effect of combined ionotropic GluR blockade in the NTS of anesthetized rats, which completely blocks the ventilatory response to carotid body stimulation (Vardhan et al. 1993). Together, these results suggest that understanding the differences between simultaneous versus individual ionotropic GluR blockade on the HVR may help explain the mechanisms of neural plasticity in the NTS with chronic hypoxia.

### Comparison with the literature

The main findings of the present study are that GluR antagonists microinjected into the NTS (1) decrease ventilation when breathing is stimulated by acute hypoxia in CON animals but not in CSH‐conditioned animals and (2) decrease ventilation during normoxia in CSH but not in CON animals (Fig. 4). In contrast, the hypercapnic ventilatory response is not significantly different either with or without GluR antagonists in the NTS in CON versus CSH‐conditioned rats (Figs. 3 and 6). Therefore, changes in the effects of GluR on the control of breathing with hypoxia appear specific to hypoxia‐related stimulation of breathing, and are not universal to any increase in ventilatory drive.

These results are not readily predictable given the effect of blocking individual GluR subtypes in the NTS of awake rats (Pamenter et al. 2014). We found that AMPAR blockade alone reduces ventilation whenever ventilation is stimulated by hypoxia (i.e., in response to acute hypoxia in CON or CSH‐conditioned rats and in CSH‐conditioned rats when normoxia is restored after chronic hypoxia), whereas NMDAR blockade alone abolishes increased ventilation during acute hypoxia in CSH‐conditioned rats but has no effects in normoxia or in CON rats. The effect of the GluR antagonist cocktail in the present study largely resembles that of AMPAR blockade alone (Pamenter et al. 2014) except the drop in ventilation during acute hypoxia with combined GluR blockade in CSH‐conditioned animals is not significant (Fig. 4). General agreement between the effects of NMDAR blockade alone and combined GluR blockade might be expected if AMPARs mediate “throughput” glutamatergic neurotransmission for the ventilatory chemoreflex and NMDARs modulate it; modulation of the HVR may not be evident if the reflex is blunted already. However, it is not clear how adding NMDAR blockade could reduce the effect of AMPAR blockade to decrease ventilation during acute hypoxia after chronic hypoxia; this effect was significant after AMPAR blockade (Pamenter et al. 2014), but not after the GluR antagonist cocktail (Fig. 4). Perhaps the activity of GluR subtypes can be upregulated via a cross‐talk mechanism to compensate for the lost activity of other local GluR subtypes that have been blocked by pharmacological antagonists as described for the hippocampus (Bai et al. 2002), and chronic hypoxia may have additional effects on such cross talk. Indeed, in anesthetized rats, simultaneous blockade of NMDARs and AMPARs abolishes the phrenic nerve response to carotid body stimulation, whereas blocking NMDARs or AMPARs alone does not affect ventilatory responses to carotid body stimulation in this preparation (Vardhan et al. 1993). Therefore, understanding the impact of simultaneous versus individual ionotropic GluR blockade on the HVR is important.

Alternatively, the effects of combined GluR blockade on the HVR before and after acclimatization to sustained hypoxia may be similar to the effects of AMPAR blockade alone and experimental error could cause the difference between CSH‐conditioned rats breathing hypoxic gas. Breathing frequency decreases significantly under such conditions by combined GluR blockade (Fig. 4) or independent AMPAR blockade (Pamenter et al. 2014). As discussed below, ventilation may not have decreased significantly with combined GluR blockade because it was limited by hypocapnia in CSH‐conditioned rats breathing hypoxic gas, that is, there is a “ceiling effect” on tidal volume ventilation before blockade so a decrease cannot be observed. While the exact mechanism of changes in the response to combined GluR blockade with CSH remain to be determined, they may involve phosphorylation of NMDA and non‐NMDA receptor subunits. We previously showed that expression of the NR1 and GluR2 subunits did not change in CSH‐conditioned rats but the phosphorylated forms of the NR1 and GluR1 subunits increased 35% and 70%, respectively, relative to control rats (Pamenter et al. 2014).

The fact that we still observe a HVR in awake rats after blocking ionotropic GluRs in the NTS contrasts with the effects of a similar GluR antagonist cocktail to block the ventilatory response to carotid body stimulation in anesthetized rats (Vardhan et al. 1993). There are other examples showing that the arousal state of an animal has a significant effect on the impact of manipulating GluRs in the NTS on chemoreflexes. For example, glutamate injections into the NTS increase blood pressure in conscious rats but decrease blood pressure in anesthetized rats (Machado and Bonagamba 1998). This could be explained by differences in cross talk between GluR types (cf. Bai et al. 2002) with arousal state, or effects of additional neurotransmitters, receptors, or synapses in the ventilatory chemoreflex arc that are activated by afferent input or independently by hypoxia. For example, excitatory dopamine type‐2 receptors (D_2_Rs) are found in the CNS and dopamine levels increase in the NTS during chronic hypoxia (Olson et al. 1983). D_2_R null mutant mice, and also wild‐type mice in which D_2_Rs are systemically inhibited, tend to have decreased 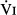, fr, and Vt during hypoxia relative to wild‐type mice (Olson and Saunders 1987; Huey et al. 2000), and VAH is prevented in D_2_R null mice (Huey et al. 2000). Dopamine release is mediated by GluR‐mediated excitation of dopaminergic neurons such that combined antagonism of NMDARs and AMPARs inhibits dopamine release in rat prefrontal cortex (Livingstone et al. 2009, 2010). Therefore, glutamate release may mediate VAH at parallel synaptic inputs to the NTS, for example, by mediation of dopamine release versus indirect activation of glutamatergic synapses between carotid body afferents and second order neurons in the NTS.

It is also conceivable that glutamate may mediate increased excitatory synaptic activity through effects on mGluRs. Typically, mGluRs at presynaptic interneurons negatively regulate presynaptic glutamate release via an interaction with GABA_B_ receptors (Fawley et al. 2011; Fernandes et al. 2011). The role of mGluRs in breathing in the NTS has not been examined, but investigation of NTS control of cardiovascular responses has shown that mGluRs in the NTS mediate a net excitatory tone and agonists of mGluRs injected into the retrotrapezoid nucleus induce sustained increases in phrenic nerve activity and this effect is dependent on prolonged glutamate release (Li and Nattie 1995). However, these effects are dependent on mGluR‐mediated excitation of NMDARs (Mueller et al. 2005), and are therefore unlikely to occur in our system where NMDARs are blocked.

Recent electrophysiological studies of NTS second order neurons in ex vivo brainstem slices provide evidence of roles for additional membrane proteins in mediating changes in neuronal excitability in CSH. For example, in neurons from nonacclimatized rats, hypoxia activates an inhibitory ATP‐sensitive K^+^ (K_ATP_) channel‐mediated conductance that opposes neuronal excitation (Zhang et al. 2008). In neurons from CSH‐conditioned rats, this current is markedly reduced, likely due to a significant reduction of K_ATP_ channel subunit expression in the NTS. This mechanism would render NTS second order neurons considerably more excitable to stimulation following acclimatization to CSH. Electrical stimulation via nonglutamatergic pathways may thus be enhanced by this postsynaptic mechanism and underlie a portion of the GluR antagonist‐insensitive ventilatory response. Signaling pathways for such mechanisms of plasticity are not known; however, CSH induces the expression of proinflammatory cytokines and chemokines in the brainstem, and concurrent treatment with ibuprofen blocks both VAH and CSH‐mediated increases in inflammatory cytokines in the brainstem (Popa et al. 2011). Changes in hypoxic‐sensitive gene expression may also be involved (Prabhakar and Semenza 2012).

### Critique of methods

A possible complication in the interpretation of our results could be incomplete blockade of GluRs in the target region or unplanned effects outside the target region. This could occur with problems in the drug dose, drug wash off, microinjection site location, or unpredictable drug diffusibility in vivo. We took numerous steps in experimental design to minimize the likelihood of such errors. First, we selected drugs that had very long washout times of many hours to days and utilized drug concentrations that were in the high end of those used in previous experiments targeting the same receptors in the same experimental preparations (Florentino et al. 1990; Ohta et al. 1993; Mizusawa et al. 1994; Machado et al. 2000). At the concentrations we utilized, these drugs have been demonstrated to effectively alter physiological responses and abolish agonist‐induced physiological responses. We opted to inject boluses of cocktail into each of the two sides of the NTS to increase the likelihood that we would saturate the targeted receptors in the NTS. However, this also increases the possibility of drug spillover outside of the NTS region and it is possible that the microinjections affected regions adjacent to the NTS to impact ventilation. The relative spread of inhibition from the cocktail in a living preparation with active circulation relative to dye injection measured postmortem is unknown but histological data suggest our effects were limited to the target region. Also, we performed tests to show our GluR manipulations blocked the effects of glutamate microinjections on ventilation (Fig. 2), which were as expected and reported previously (e.g., Vardhan et al. 1993; Mizusawa et al. 1994; Costa and Garlid 2008; Pamenter et al. 2014). Hence, our conclusions that the effect of combined ionotropic glutamate receptor blockade is different than that predicted from independent blockade of NMDA or AMPA receptors must be qualified. This conclusion assumes that small spatial deviations in the location of microinjections and in exact neurons being affected in the different preparations are not physiologically significant. However, greater precision is impossible in these experiments on awake animals, which require stereotaxic surgery based on external landmarks that can vary between individuals, and physiological responses (e.g., glutamate induced increases in breathing) that may not identify unique respiratory neurons.

Finally, the effects we observe of GluR antagonists on breathing cannot be explained by changes in arterial blood gases. In general, Pao_2_ and Paco_2_ decrease in animals breathing hypoxic gas mixtures due to decreased ambient oxygen availability and hypoxia‐induced hyperventilation, respectively (Smith et al. 2001). Similarly, Paco_2_ decreases in acclimatized CSH‐conditioned animals with time‐dependent increases in ventilation, in good agreement with previous studies (Smith et al. 2001). In our experiments, GluR antagonism did not have significant effects on Pao_2_ or Paco_2_ in any experimental group, indicating that any differences in ventilatory responses are not explained by significant differences in chemoreceptor stimuli. Any trends for changes in blood gases with drugs were generally as expected (Fig. 7). For example, chronic hypoxia increases ventilation and decreases Paco_2_. Paco_2_ would be predicted to increase more with the significant decrease in 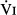 in CSH‐conditioned animals breathing 21% O_2_ than in CON animals breathing 10% O_2_ because hyperventilation in 10% O_2_ moves the operating point on the hyperbola relating alveolar Pco_2_ to ventilation. Furthermore, decreases in metabolic rate with acute hypoxia in laboratory rodents can mask the effects of changes in ventilation on arterial blood gases. Finally, however, there may be some effect of blood gases on the ventilatory values in the CSH‐conditioned animals breathing 10% O_2_. The very low Paco_2_ in these conditions may limit hyperventilation without drug, such that 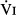 is already low without the drug and does not decrease further (or significantly) with the drug. This would not reflect a mechanical limitation because 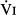 can increase to higher levels breathing CO_2_ (cf. Fig 6) but rather a limitation of the control system that must integrate arterial Po_2_ and Pco_2_ regulation.

### Perspectives and significance

Our results demonstrate a critical role for glutamate receptor‐dependent plasticity in the NTS in ventilatory acclimatization to hypoxia, which may be the result of several redundant signaling pathways. For example, dopamine and glutamate release at the NTS may each contribute to VAH and one pathway may be capable of compensating for decreases in the other. In this case, the NMDAR‐dependent component of VAH is not manifest when AMPAergic throughput neurotransmission in the NTS is blocked; however, dopaminergic inputs may become paramount when total NTS glutamatergic signaling is abrogated, leading to no observable effect of GluR antagonism at the NTS on ventilation in CSH‐acclimatized animals. This hypothesis could be tested in future experiments by simultaneous blockade of NTS D_2_Rs and GluRs to determine whether such cotreatment entirely abolishes responses to hypoxia in both CON and CSH‐conditioned animals. This excitatory/inhibitory balance is further confounded by cross talk between GluRs (Bai et al. 2002), and it is unclear what effect blockade of ionotropic glutamatergic signaling would have on the net balance of metabotropic signaling at mGluRs. This highlights the complexity of synaptic signaling in the hypoxic ventilatory response, and possibilities for mechanisms of plasticity in the ventilatory chemoreflexes. Elucidating such cellular mechanisms of plasticity may inform therapeutic treatments for pathologies related to chronic hypoxemia.

## Conflict of Interest

None declared.
